# Tribological Behavior and the Mild–Severe Wear Transition of Mg97Zn1Y2 Alloy with a LPSO Structure Phase

**DOI:** 10.3390/ma11040505

**Published:** 2018-03-27

**Authors:** Wei Sun, Xihua Xuan, Liang Li, Jian An

**Affiliations:** 1 Key Laboratory of Automobile Materials, Ministry of Education, Jilin University, Changchun 130025, China; swyx@ciac.jl.cn (W.S.); liangliang-2005@163.com (L.L.); 2 Department of Materials Science and Engineering, Jilin University, Changchun 130025, China; 3 Chery Jaguar Land Rover Automotive Co., Ltd., Changshu 215513, China; Xuanxihua434@163.com

**Keywords:** magnesium alloys, tribological behavior, wear maps, mild-severe wear transition, dynamic recrystallization

## Abstract

Dry friction and wear tests were performed on as-cast Mg97Zn1Y2 alloy using a pin-on-disc configuration. Coefficients of friction and wear rates were measured as a function of applied load at sliding speeds of 0.2, 0.8 and 3.0 m/s. The wear mechanisms were identified in the mild and severe wear regimes by means of morphological observation and composition analysis of worn surfaces using scanning electron microscope (SEM) and energy dispersive X-ray spectrometer (EDS). Analyses of microstructure and hardness changes in subsurfaces verified the microstructure transformation from the deformed to the dynamically recrystallized, and properties changed from the strain hardening to dynamic crystallization (DRX) softening before and after the mild–severe wear transition. The mild–severe wear transition can be determined by a proposed contact surface DRX temperature criterion, from which the critical DRX temperatures at different sliding speeds are calculated using DRX dynamics; hence transition loads can also be calculated using a transition load model. The calculated transition loads are in good agreement with the measured ones, demonstrating the validity and applicability of the contact surface DRX temperature criterion.

## 1. Introduction

Among magnesium alloys, Mg97Zn1Y2 alloy has attracted a lot of attention due to its unique long-period stacking ordered (LPSO) structure phase [1,2]. The LPSO structure phase in Mg97Zn1Y2 alloy is typically an intermetallic compound X-Mg_12_ZnY with a long-period 18 R modulated structure [3]. The most famous role of the X-Mg_12_ZnY phase was found to be a strengthening phase in an Mg97Zn1Y2 alloy prepared via a rapidly solidified powder metallurgy (RS/PM) technique in 2011. The RS/PM Mg97Zn1Y2 alloy demonstrates extraordinary mechanical properties at room and elevated temperatures, namely high yield strength above 600 MPa and elongation of 5% at room temperature and yield strength of 510 MPa at 150 °C [4]. Moreover, Mg97Zn1Y2 alloy also displays other excellent characteristics, which makes it greatly different from those conventional magnesium alloys containing ordinary intermetallic compound phases. X-Mg_12_ZnY phase with LPSO structure enhances strength by preventing the growth of {10–12} deformation twin in Mg matrix [5], meanwhile it contributes significantly to ductility by deforming through the kink band mechanism when subjected to compressive stress parallel to the (0001) plane [6]. Furthermore, the LPSO phase exhibited a rather high thermal stability up to 500 °C in an extruded Mg97Zn1Y2 alloy, which was proved by the unchanged morphology of the LPSO phase even after an annealing treatment at the elevated temperature of 500 °C [7].

Generally speaking, conventional magnesium alloys such as AZ (Mg-Al-Zn), AM (Mg-Al-Mn) alloys are not considered suitable for tribological applications owing to their relatively low mechanical properties except when they are reinforced with CNTs (Carbon Nanotubes) or ceramic particles [8,9]. However, the emergence of high-performance Mg97Zn1Y2 alloy means it has potential in certain tribological situations which have been suitable for aluminum alloys before, such as low-load bearing gears, clutch pistons, and self-lubricating aluminum-based bearing alloys. Before promoting the tribological applications of Mg97Zn1Y2 alloy, firstly, the essential friction and wear characteristics must be well understood, such as the effects of the applied load and sliding speed on the coefficient of friction, wear rate and wear mechanisms. Secondly, the most important “safe wear operation region” for engineering applications must be determined, and a corresponding practical prediction method should also be established. The “safe wear operation region” normally refers to the mild wear regime that was derived from a notion described by Chen and Alpas in their study on the dry sliding wear behavior of AZ91 alloy [8]. They clarified the dry sliding wear behavior of AZ91 alloy into mild and a severe wear regimes. Under the mild wear regime, the surfaces of magnesium alloys suffered from only slight damage, and wear proceeded steadily with a low wear rate, whereas under the severe wear regime—i.e., “unsafe wear operation region”—the surfaces were badly damaged by severe plastic deformation or surface melting, and wear went on unsteadily because the wear rate was rather high and increased rapidly with the wear period. Numerous follow-up research studies by others also confirmed the clarification applicable to most magnesium alloys. However, to our knowledge, although the friction and wear characteristics of the most commonly used AZ, AM alloys such as AZ31, AZ51, AZ91, AM50B and AM60B alloys have been already well understood [10,11,12,13], only little is known about wear the performance of Mg97Zn1Y2 alloy containing a LPSO phase. Our previous study found that Mg97Zn1Y2 alloy exhibited superior wear resistance to AZ91 alloy due to the higher thermal stability of the LPSO phase and mechanical properties at elevated temperature [12]. However, as the previous study only focused on the wear behavior of the Mg97Zn1Y2 alloy at a constant sliding speed of 0.785 m/s, the critical conditions for the mild–severe wear transition have not been elucidated at other sliding speeds. Therefore, a safe wear operation region for Mg97Zn1Y2 alloy has not yet been provided for reference regarding the tribological application of wear components made from the alloy.

In the present work, the variations of coefficient of friction and wear rate of Mg97Zn1Y2 alloy with applied loads were measured under three typical sliding speeds—including low, intermediate and high sliding speeds—and the wear mechanisms involved were identified by scanning electron microscopy (SEM) and energy dispersive X-ray spectrometer (EDS). In addition, the safe wear operation region for tribological applications of Mg97Zn1Y2 alloy was determined by the establishment of a wear transition map using additional wear test data. The decisive mild–severe wear transition mechanism was also discussed in terms of the microstructure and hardness changes of the surface layer materials of wear specimens. An evaluation method for the mild–severe wear transition load was proposed based on a contact surface DRX criterion and identified experimentally.

## 2. Materials and Methods

A cylindrical ingot of Mg97Zn1Y2 (in atomic percentage) alloy was prepared by using conventional gravity casting process from pure Mg (99.9 wt %), Zn (99.9 wt %) and Mg-20.3 wt % Y master alloy in an electrical furnace under a shielding gas of CO_2_-0.05%SF_6_. The ingot had the dimensions of diameter 95 mm and length 200 mm, from which specimens of 8 mm diameter and 12 mm length were directly machined for measurements of hardness and compressive properties. The phase constituents in the alloy were analyzed by a Rigaku D/MAX 2500PC X-ray diffractometer (XRD, Tokyo, Japan). The optical microstructure of Mg97Zn1Y2 alloy was observed using a LEXT-OLS3000 confocal scanning laser microscope (Orangeburg, NY, USA) after polishing and etching in a picric acetic solution.

Friction and wear tests were performed on a MG2000 pin-on-disc tribometer. All friction and wear tests were conducted at a room temperature of 20 °C under dry sliding wear condition. The pins of 6 mm diameter and 13 mm length were machined out from the Mg97Zn1Y2 alloy ingot, and polished and thoroughly degreased by acetone and dried before the commencement of each wear test. The discs of 70 mm in diameter and 10 mm in thickness were made of AISI/SAE5150 steel with a quenching hardness of 57 HRC. The diameter of wear tracks on the discs were 60 mm. The friction and wear tests were carried out at three selected typical sliding speeds for 377 m (3000 cycles) to investigate the wear behavior in detail. These were low, intermediate and high sliding speeds of 0.2, 0.8 and 3.0 m/s, respectively. In order to accurately establish a wear mechanism transition map and demarcate the safe wear operation region for engineering applications, friction and wear tests were also carried out at the additional sliding speeds of 0.5, 1.0, 2.0 and 4.0 m/s. Prior to each wear test, the surfaces of the pins and discs were polished to a constant surface roughness of about 0.4 μm Ra and cleaned in an acetone solution. The friction moments and coefficients of friction were measured continuously with an electronic sensor attached to the testing machine. The wear rate was calculated through the volume loss divided by the sliding distance. The volume wear loss was determined by the height difference of the pin after a sliding wear test using a digital precision micrometer with an accuracy of 0.001 mm. The worn surface morphologies were examined by a Carl Zeiss-EVO18 scanning electron microscope (Oberkochen, Germany) equipped with an LINK-ISIS energy dispersive X-ray spectrometer. The cross-sectional microstructures of subsurfaces parallel to the sliding direction were observed after wear tests under a LEXT-OLS3000 confocal scanning laser microscope, and the Vickers hardness of the surbsurfaces was measured as a function of depth from the surface by a HVS-1000 microhardness tester using a load of 0.49 N for a dwell period of 15 s.

## 3. Results and Discussions

### 3.1. Microstructure and Mechanical Properties

**Figure 1:**
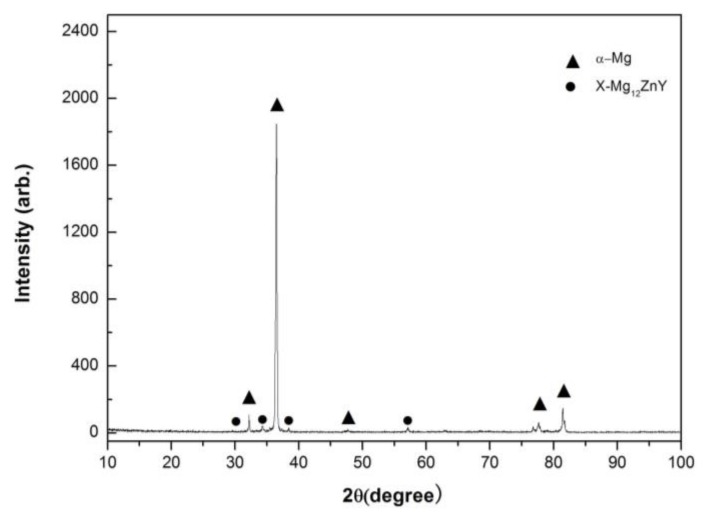
XRD (X-ray diffraction) spectrum of Mg97Zn1Y2 alloy.

**Figure 2:**
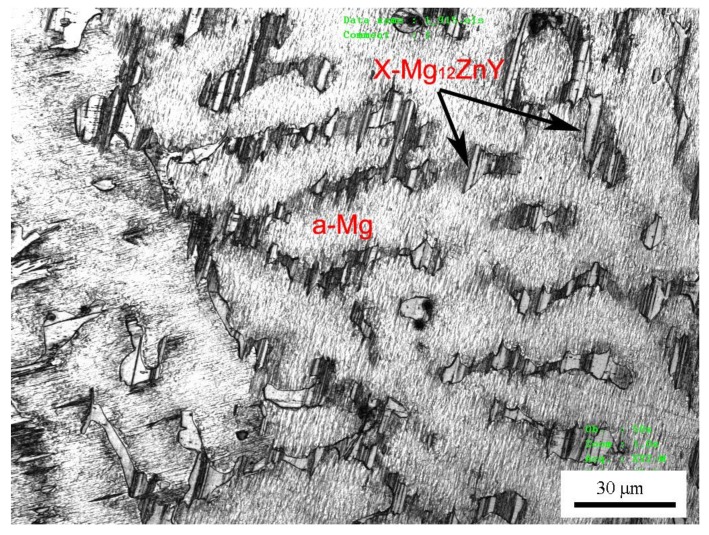
Optical microstructure of Mg97Zn1Y2 alloy.

The X-ray diffraction (XRD) analysis of the Mg97Zn1Y2 alloy is shown in Figure 1. This pattern indicates that the alloy consists of α-Mg solid solution phase and X-Mg_12_ZnY compound phase. The XRD peaks of the X-Mg_12_ZnY phase in Figure 1 agree with those reported in as-cast and hot-rolled Mg97Zn1Y2 and Mg96Zn1Y3 alloys [14,15,16]. Therefore, the X-Mg_12_ZnY phase in the studied alloy has a LPSO structure. The optical microphotograph of the Mg97Zn1Y2 alloy is illustrated in Figure 2. The microstructure exhibits typical coarse dendrites of α-Mg phase surrounded with a network of a X-Mg_12_ZnY eutectic phase. The measured compressive properties and Vickers hardness of Mg97Zn1Y2 alloy are listed in Table 1.

### 3.2. Coefficients of Friction and Wear Rates 

**Figure 3:**
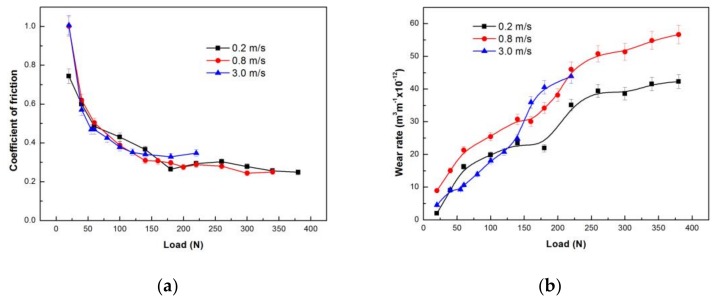
Coefficients of friction (**a**) and wear rates (**b**) as a function of applied load for tests conducted at different speeds of 0.2, 0.8 and 3 m/s.

The variations of the coefficient of friction and wear rate with applied load at the three selected sliding speeds are shown in Figure 3. Under the three given sliding speeds, the coefficient of friction curves exhibit a similar decreasing and maintaining trend with an increasing load, as shown in Figure 3a—i.e., they all decrease rapidly at first when load is not higher than 60 N or 55 N, then reduce a little gently, and finally maintain low values with further increasing load. However, there are still some differences between them. At the low sliding speed of 0.2 m/s, the coefficient of friction decreased remarkably within 20–60 N, and decreased almost lineally within 60–180 N, then rose a little at 220 N before entering a stable stage of 0.25–0.3 until 380 N. At the intermediate and high sliding speeds of 0.8 and 3.0 m/s, both coefficients of friction also decreased considerably within 20–60 and 20–55 N respectively, then in case of 0.8 m/s, the coefficient of friction reduced slightly within 60–160 N and finally decreased a little to a low level of 0.24–0.28 till 380 N, while in case of 3.0 m/s, the coefficient of friction decreased the least within the load range of 55–120 N, and finally entered a stable stage of 0.32–0.35 until 220 N. It is noted that among the three stable stages of the coefficient of friction, the highest was at the high sliding speed of 3.0 m/s, while the others were almost equal.

The variations of wear rate with load also showed clear stage features at the three given sliding speeds, and they were more pronounced than the variations of coefficient of friction, as shown in Figure 3b. At 0.2 m/s, there were two distinct stages: the first stage was within 20–180 N, where the wear rate increased gradually, and the second stage was within 180–380 N, where the wear rate also increased slowly with the applied load except for a sudden increase at 220 N. At 0.8 m/s, there were three stages: the wear rate increased rapidly with the increasing load at the first stage within 20–60 N, then increased gradually at the second stage within 60–160 N, and finally rapidly increased to a high level within the third stage of 160–380 N. At 3.0 m/s, there were also three stages; the wear rate increased gently at the first stage within 20–55 N, then increased quickly at the second stage within 60–140 N, and finally entered a high level stage within 140–220 N. It was found that the wear rate was higher at 0.8 m/s than at 0.2 m/s within the whole applied load range of 20–380 N, while the wear rate was almost the lowest at 3.0 m/s within 20–120 N, and then it rose rapidly and exceeded the two others within 160–220 N. It is also noticeable that the variation stages of coefficients of friction in general corresponded to those of wear rates, suggesting that wear should be controlled by different mechanisms within different stages. Therefore, worn surface morphologies were observed and analyzed using SEM and EDS techniques to identify the wear mechanisms under different sliding conditions.

### 3.3. Wear Mechanisms

**Figure 4:**
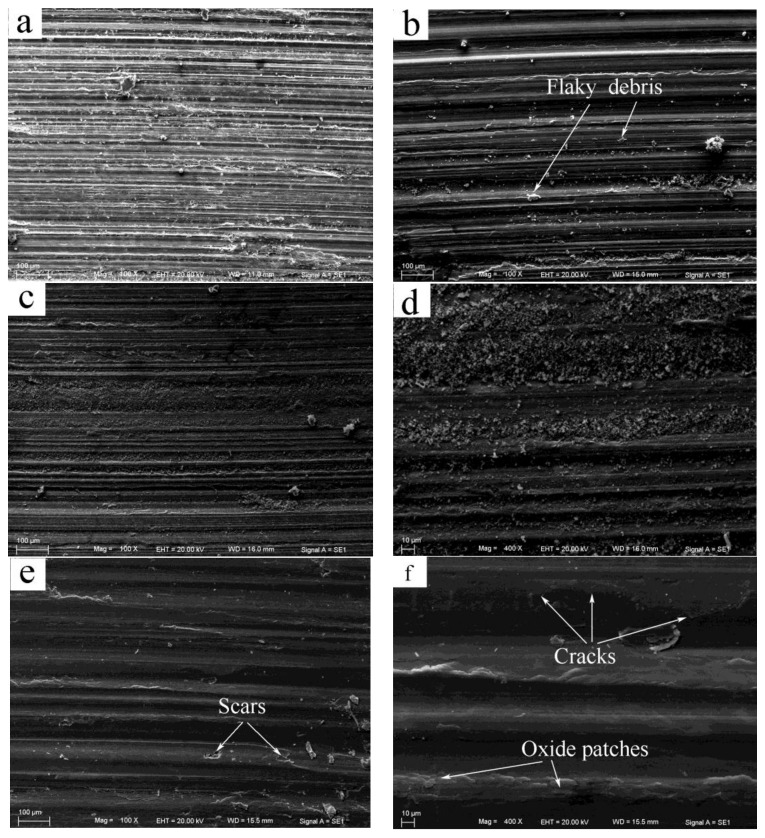
SEM (scanning electron microscope) morphologies of worn surfaces of Mg97Zn1Y2 alloy at 0.2 m/s and different applied loads: (**a**) 20 N, (**b**) 100 N, (**c**) 180 N, (**d**) 180 N, (**e**) 260 N, (**f**) 260 N.

SEM micrographs of the worn surfaces subjected to different loads at the low sliding speed of 0.2 m/s are depicted in Figure 4. The worn surfaces were firstly observed for pins worn at the first stage of the wear rate curve. At 20 N, the worn surface presented typical features of abrasion and oxidation wear, which were characterized by a series of deep grooves parallel to the sliding direction and a large amount of fine scattered debris, as shown in Figure 4a. EDS revealed that the oxygen element content on the worn surface was as high as 11.26%. Since the deep grooves exhibited minimal displacement of material to the sides, the type of deep grooves was apparently formed by microcutting of hard asperities of the disc or detached particles from the pin. This suggests that the microcutting mechanism has a dominant effect on the friction force, resulting in a high coefficient of friction of 0.74. With the increasing load to 100 N, the grooves showed a wide displacement of material to the side, and some flaky debris was found on the worn surface, as shown in Figure 4b; meanwhile the oxygen element content decreased to 8.57%. These attributes demonstrate that abrasion was still the main wear mechanism, but the micro mechanism was transformed from microcutting to ploughing, and oxidation wear also accompanies abrasion wear. As the load was increased to 180 N, the worn surface morphology changed greatly. The depth and number of grooves decreased significantly, but the oxidation wear climaxed, because a great deal of fine powders debris detached from the oxidation layer was left all over the worn surface, as shown in Figure 4c. The oxygen element content on the worn surface reached a high level of 15.7%. From the magnification microphotograph shown in Figure 4d, a large scale of thin oxidation layer was found breaking into fine powders on the worn surface. However, when the load exceeded 180 N, i.e., at the second stage of the wear rate curve, the wear mechanism changed into a totally different type. At 260 N, the worn surface exhibited a certain extent of plastic deformation with a flattened surface, and a few irregular scars were formed together with several incidents of sheet debris on the worn surface, as shown in Figure 4e. A few cracks were observed perpendicular to or at a certain angle to the sliding direction on the worn surface in the magnification microphotograph shown in Figure 4f. This is a typical feature of delamination wear. In addition, the oxygen element content of the worn surface was found to increase to the highest level of 19.4%, but no evident spallation of the oxide layer was observed except for small oxide patches formed on the localized area, suggesting that the main wear mechanism was a mixed mode of delamination and heavy surface oxidation.

The SEM analysis of the worn surfaces can explain the lowest coefficient of friction at 180 N and the rapid increase of the wear rate above 180 N that occurred in Figure 3a,b, respectively. It is the presence of a large amount of fine oxide powder between the contact surfaces that might have significantly helped reduce the coefficient of friction to the low value of 0.26, and maintain a steady state with a low wear rate even lower than the value at 140 N. Therefore, when the fine powders disappeared from the worn surface at loads above 180 N, a rapid increase in the wear rate occurred when the wear was controlled by the delamination mechanism. Apparently, the rapid rising of the wear rate above 180 N does not correspond to the mild–severe wear transition, since the delamination that occurred above 180 N is a typical wear mechanism in a mild wear regime. The SEM examination of the worn surfaces identified that under 0.2 m/s, the wear proceeded in the mild wear regime throughout the experimental load range of 20–380 N. This conclusion is similar to the experimental results of AZ, AM and AS alloys, which also showed mild wear behavior when tested at the low sliding speeds of 0.1 and 0.2 m/s [8,10,11,12,13].

**Figure 5:**
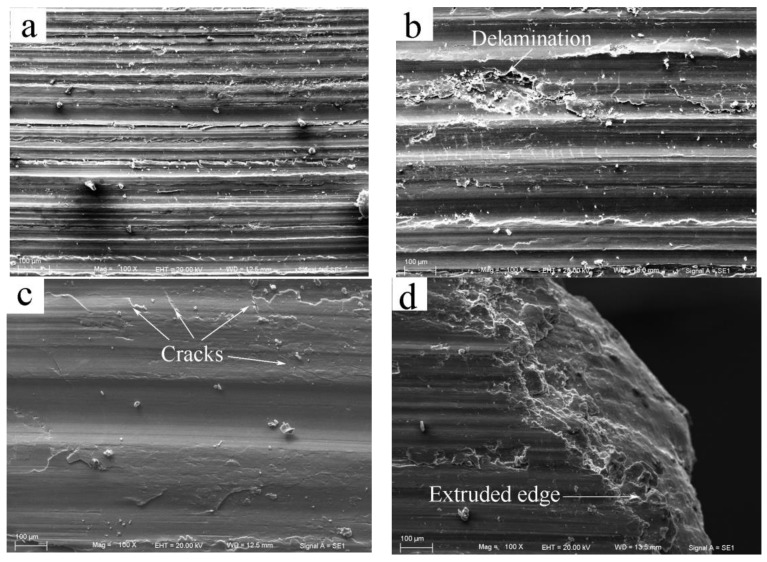

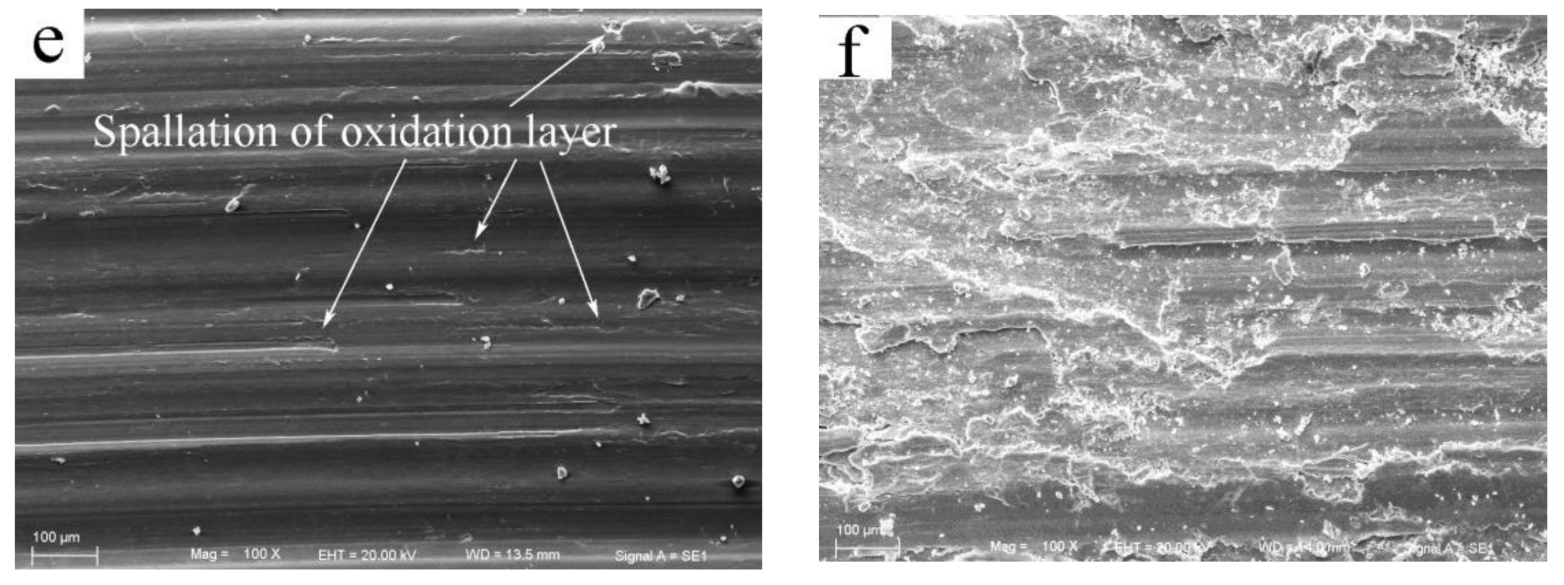
SEM morphologies of worn surfaces of Mg97Zn1Y2 alloy at 0.8 m/s and different applied loads: (**a**) 20 N, (**b**) 60 N, (**c**) 140 N, (**d**) 260 N, (**e**) 260 N, (**f**) 380 N.

SEM images of the worn surfaces at the intermediate sliding speed of 0.8 m/s are shown in Figure 5. At the first stage of the wear rate curve, when the applied load was 20 N, the worn surface also exhibited the features of abrasion wear and oxidation wear—i.e., a number of grooves and fine debris were produced on the worn surface, as shown in Figure 5a—and meanwhile, the oxygen element content of the worn surface was 18.20%. With the increased load to 60 N, wear entered the second stage, and the wear mechanism started transforming to delamination and heavy surface oxidation, because several irregular scars and a series of cracks perpendicular to the sliding direction were observed in Figure 5b. In addition, the oxygen element content of the worn surface was still maintained high above 10%. The delamination mechanism became much more evident at 140 N since cracks emerged extensively on the worn surface, and the plastic deformation extent of surface layer material increased, as shown in Figure 5c. When the load was increased above 160 N, wear entered the third stage. For example, at 260 N, the surface was apparently subjected to a severe plastic deformation since the surface layer material was extruded out at the edge of pin, and a few pieces of oxidation layer were found detached at the center of the worn surface, as shown in Figure 5d,e. Since the oxygen element content was still maintained above 13%, the oxidation layer was largely not destroyed. The wear mechanism was a mixed mode of severe plastic deformation and spallation of the oxidation layer. The onset of severe plastic deformation actually indicated a mild–severe wear transition, which resulted in the rapid increase of the wear rate at 180 N. At the largest experimental load of 380 N, the spallation of oxidation layer became much more severe, all over the worn surface, as shown in Figure 5f. Therefore, this type of mixed wear mechanism can prevail through the load range of 160–380 N.

**Figure 6:**
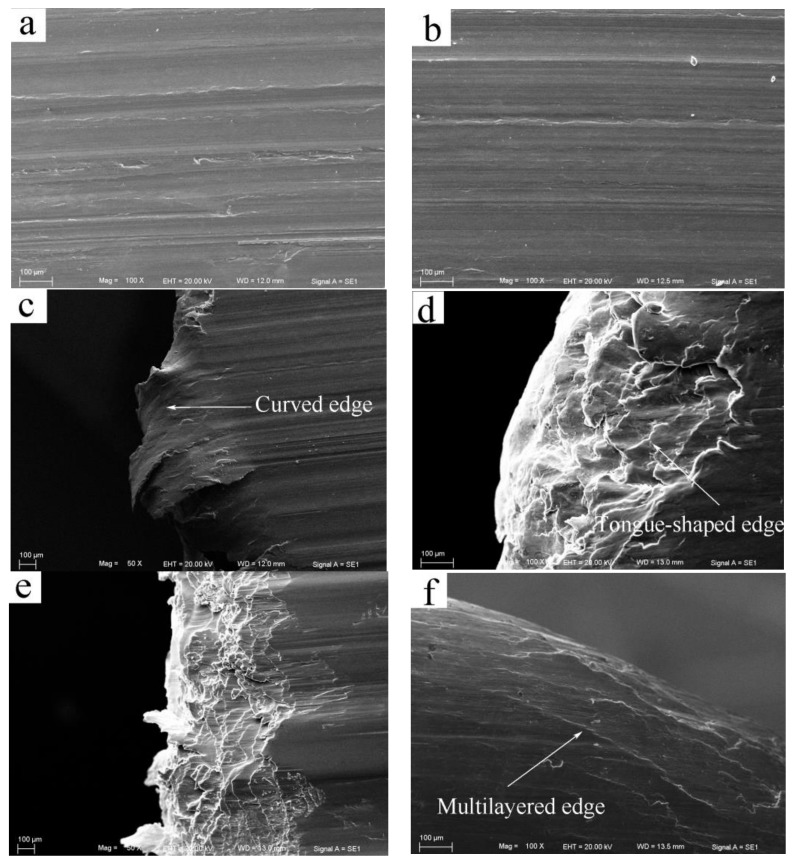
SEM morphologies of worn surfaces of Mg97Zn1Y2 alloy at sliding speed of 0.3 m/s and 4.0 m/s and different applied loads: (**a**) 20 N, (**b**) 60 N, (**c**) 100 N, (**d**) 180 N, (**e**) 260 N, (**f**) 140 N, 4 m/s.

For the high sliding speed of 3.0 m/s, the SEM micrographs of the worn surfaces are shown in Figure 6. When wear was at the first stage, at 20 N, the worn surface was flattened due to a certain extent of plastic deformation, and a few irregular scars were observed, as shown in Figure 6a. An important point to note is that the oxygen content of worn surface is only 6.7%, suggesting that the surface oxidation layer cannot maintain its extensive existence at such a high sliding speed. Therefore, the main wear mechanism was delamination. When the load was increased above 55 N, wear entered the second stage. For instance at 60 N, the worn surface was severely deformed, leaving behind a smooth surface without cracks, as shown in Figure 6b. This represents the commencement of the mild–severe wear transition. The plastic deformation extent was more evident at 100 N because the surface layer material was largely extruded out of the contact surface forming a curved edge, as shown in Figure 6c. Even so, the wear rate was found not to exceed those subjected to equal loads under the sliding speed of 0.2 and 0.8 m/s in the mild wear regime. This rare phenomenon was not found during the investigation of the wear behavior of AZ and AS alloys. Once the wear behavior of the AZ and AS alloys transited to severe wear at the sliding speed of 3.0 m/s, the wear rate would immediately surpass those in the mild wear regime under 0.1 and/or 0.8 m/s [8,11,17]. The smaller rise in the wear rate for Mg97Zn1Y2 alloy after the mild–severe wear transition, could be attributed to a smaller drop of surface hardness compared with AZ and AS alloys. The worn surface hardness of Mg97Zn1Y2 alloy was measured at the transition load and plus 25 N—i.e., at 55 N and 80 N using a microhardness tester. The wear rate increase and surface hardness decrease of Mg97Zn1Y2 alloy at the two applied loads are listed in Table 2 for comparison with the experimental results for AZ and AS alloy in references [10,11,17]. It can be seen that as the applied load is increased to 25 N above the transition load, the worn surface hardness of the Mg97Zn1Y2 alloy decreased from 134 HV to 125 HV, only a 7.5% reduction ratio, which is the lowest among the four given types of magnesium alloys. However, AZ31, AZ51 and AS31 alloys exhibit a surface hardness reduction ration above 10% when subjected to an applied load 20 N higher than the transition load, where the AZ31 alloy has the largest reduction ratio value of 13.6%. When further increasing the load to 180 N—i.e., when wear entered the third stage—a sign of surface melting was observed since the worn surface was much smoother than that under the severe plastic deformation mechanism, and a tongue-shaped edge was formed, as shown in Figure 6d. Surface melting is a typical wear mechanism for magnesium alloys that is induced by a large frictional heating, and has been observed in many magnesium alloys, such as AZ31, AZ51, AZ91, Mg-Al-Si alloys, under high loads and/or high sliding speeds [7,8,9,15]. Even when the load was increased to 260 N, the edge still maintained the tongue shape as shown in Figure 6e, rather than the frequently-reported multilayered structural edge of AZ alloys. This could be ascribed to the fact that the Mg97Zn1Y2 alloy has a much higher melting temperature (about 543 °C) than AZ alloys (about 431 °C) [14], therefore the surface layer material did not completely melt at loads under 3.0 m/s. Only at a higher sliding speed, such as 4.0 m/s, did the morphology of the surface melting mechanism present a typical multilayered structure edge with the help of larger frictional heating, as shown in Figure 6f.

### 3.4. Wear Rate and Wear Transition Maps

It is well known that wear proceeds steadily for magnesium alloys under wear mechanisms such as oxidation, abrasion and delamination wear [8,10,11,12,13]. The volumetric wear increases slowly in a linear relationship with the sliding distance, and wear surfaces are damaged slightly and are generally covered by tribological layers. These wear mechanisms of magnesium alloys are included in mild wear regimes according to the notation proposed by Archard and Hirst [18]. In contrast, the wear progresses in an unsteady state have a higher wear rate for magnesium alloys under wear mechanisms including severe plastic deformation and surface melting. These two wear mechanisms are classified as severe wear, since the wear surfaces are massively damaged, and are usually subjected to large-scale material transfer to the counterface. 

**Figure 7:**
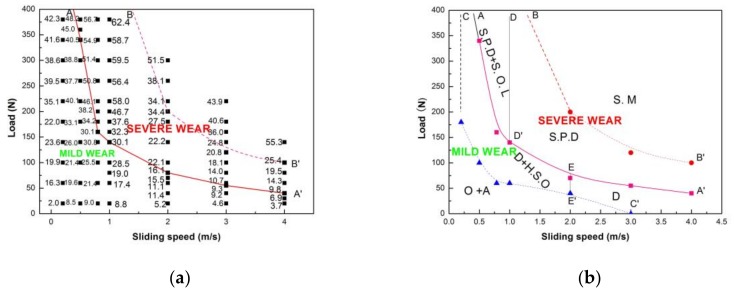
Wear rate map (**a**) and wear transition map (**b**) for Mg97Zn1Y2 alloy. O-Oxidation; A-Abrasion; D-Delamination; H.S.O-Heavy Surface Oxidation; S.P. D-Severe Plastic Deformation; S.O.L-Spallation of Oxide Layers; S. M-Surface melting.

In order to provide a useful reference for the tribological engineering application of Mg97Zn1Y2 alloy, the boundary between the mild and severe wear regimes was carefully drawn by conducting additional friction and wear tests at other sliding speeds including 0.5, 1.0, 2.0 and 4.0 m/s, and corresponding analyses were carried out based on SEM and EDS examinations of the worn surfaces. The chemical compositions of the worn surfaces were examined by EDS and listed in Table 3. The wear rate map of the Mg97Zn1Y2 alloy was constructed on a rectangular coordinate system with applied load as the vertical coordinate and sliding speed as the horizontal coordinate, as shown in Figure 7a. The wear rate data were obtained under different loading conditions at sliding speeds of 0.2–4.0 m/s, and they are presented in 10^−12^ m^3^m^−1^ on the map. SEM observations and the chemical composition information of the worn surfaces were utilized to draw the boundary lines separating the wear regimes and sub-regimes. It can be seen that increasing sliding speed decreases the wear rate at the boundary between the mild and severe regimes, and that the wear rate decreases faster at the low and intermediate sliding speeds of 0.5–2.0 m/s than at the high sliding speed of 3.0–4.0 m/s. For example, the wear rates at transition boundary were 40.5 × 10^−12^ m^3^m^−1^ at 0.5 m/s, 30.1 × 10^−12^ m^3^m^−1^ at 0.8 m/s and 1.0 m/s, and 15.5 × 10^−12^ m^3^m^−1^ at 2.0 m/s, however only 10.7 × 10^−12^ m^3^m^−1^ at 3.0 m/s and 9.8 × 10^−12^ m^3^m^−1^ at 4.0 m/s, respectively. This means that the wear rate data cannot fully reflect the real extent of the surface damage, especially at high sliding speeds, because the extensive surface damage mechanisms originating from the severe plastic deformation and surface melting are not considered by the volumetric wear rate.

The wear transition map for Mg97Zn1Y2 alloy is shown in Figure 7b. The map can be classified into two main wear regimes, i.e., the mild wear regime (safe wear operation region) and the severe wear regime. They are separated by solid line AA′, i.e., below AA′ is the mild wear regime, and above AA′ is the severe wear regime. At 0.2 m/s, the mild–severe wear transition does not occur until the largest experimental load of 380 N. Increasing the sliding speed from 0.5 to 4.0 m/s decreases the transition load from 340 N to 40 N in the exponential decay mode. The mild wear regime consists of three sub-regimes, namely oxidation + abrasion, delamination + heavy surface oxidation and delamination. The severe wear regime is composed of three sub-regimes, i.e., severe plastic deformation + spallation of oxidation layer, severe plastic deformation and surface melting. The transition boundary AA′ was established mainly according to the onset of severe plastic deformation. The wear transition from severe plastic deformation to surface melting is indicated by the dashed line BB′; above the line BB′ is the surface melting sub-regime. The boundary line CC′ between the oxidation + abrasion sub-regime and the delamination + heavy surface oxidation and delamination sub-regimes was determined by the occurrence of delamination mechanisms and/or disappearance of abrasion mechanisms. The boundary line DD′ between severe plastic deformation + spallation of oxidation layer and severe plastic deformation sub-regimes was determined by the occurrence of spallation of the oxidation layer by SEM and the existence of a high content of oxygen elements by EDS on the worn surfaces. The boundary line EE′ between delamination + heavy surface oxidation and delamination sub-regimes was determined by the high oxygen element content in the range of 7.53–20.42% on the worn surfaces subjected to delamination wear.

### 3.5. Microstructure Change Mechanism for the Mild–Severe Wear Transition

From the wear transition map, it can be seen that increasing the sliding speed decreases the mild–severe wear transition load; the transition load decreases most rapidly at low and intermediate sliding speeds within 0.5–1.0 m/s. In addition, it was found from the morphological analysis of the worn surfaces that just before the wear transition, the main wear mechanism was delimination or delimination + surface oxidation. In the delamination mechanism, the cracks initiate beneath the surface and propagate to the surface, finally causing the removal of material in the sheet shape. However, after the onset of the mild–severe wear transition, the wear mechanism transforms to severe plastic deformation, in which cracks are rarely observed and the surface layer material is extruded out the contact surface in the curved shape, especially at high sliding speed. This implies that the ductility of the surface layer material must be improved by a friction-induced microstructure change accompanying a severe plastic deformation transition. Therefore, a comparative microstructure analysis of the subsurface material was carried out between several selected specimens tested at 0.8 and 3.0 m/s.

**Figure 8:**
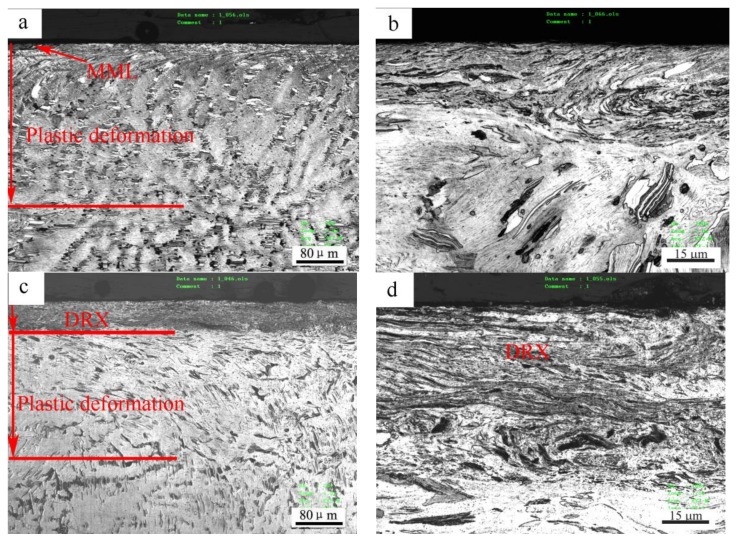
Cross-sectional optical microstructures of Mg97Zn1Y2 alloy after sliding at 0.8 m/s and different loads: (**a**) 20 N, (**b**) 100 N, (**c**) 200 N, (**d**) 200 N, showing fine DRX (dynamic crystallization) microstructure in surface layer.

The microstructure change in the subsurfaces before and after the mild–severe wear transition under 0.8 m/s is shown in Figure 8. At 140 N under the mild wear regime, the subsurface was apparently subjected to a certain extent of plastic deformation, and a plastic deformation zone was formed with a depth of about 260 μm (Figure 8a), where the dendrites elongated towards the surface. The strips of Mg_12_ZnY phase also bent in the sliding direction in the magnification microphotograph (Figure 8b). At 200 N, under the severe wear regime, a distinct fine microstructure zone of about 50 μm thickness formed above the plastic deformation zone of about 170 μm thickness (Figure 8c). This zone was actually composed of DRX microstructures because the deformed α-Mg dendrites in the top part of the plastic deformation zone were replaced by newly-formed fine grains on the submicrometer scale, despite the fact that the DRX grains were not well recognized due to the limited resolution of the microscope. The phenomenon whereby the surface material was transformed from the deformed to the DRX microstructure during dry sliding wear was also found in other magnesium alloys (AZ31, AZ51 and Mg-3Al-0.4Si), pure magnesium and pure copper, when the applied load or sliding speed surpassed a certain critical value [10,11,17,19,20]. Chen et al. [21] found a worn subsurface layer consisting of a DRX microstructure formed beneath the top nanostructured mixing layer for Cu-Al alloys, and an extremely fine DRX microstructure at the nanometer scale in subsurface layers when the Al content was higher than 1.5%. However, the Mg_12_ZnY phase in the DRX zone was found to demonstrate a strong microstructural stability, elongating in the sliding direction, but still maintaining the strip shape, and most of Mg_12_ZnY strips took positions parallel to the sliding direction (Figure 8d). The Mg_12_ZnY phase in the surface layer demonstrated a unique deformation behavior that is obviously different from those intermetallic phases in the AZ and AS alloys. The intermetallic phases, such as Mg_17_Al_12_ in AZ alloys and Mg_2_Si in AS alloys, are usually broken into particles in the surface layers even at low loads such as 20 N and 40 N [11,17]. Therefore, the Mg_12_ZnY phase could continuously act as a fiber enforcement in the surface material even after the mild–severe wear transition.

**Figure 9:**
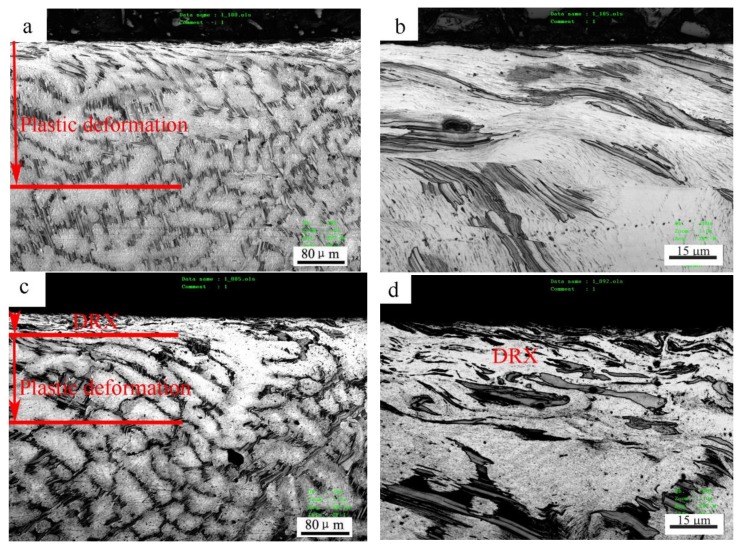
Cross-sectional optical microstructures of Mg97Zn1Y2 alloy after sliding at 3.0 m/s and different loads: (**a**) 40 N, (**b**) 40 N, showing deformation kinks of Mg_12_ZnY phase strips, (**c**) 100 N, (**d**) 100 N, showing fine DRX microstructure in surface layer.

Under 3 m/s, the surface layer also experienced a similar microstructure development before and after the mild–severe wear transition, as shown in Figure 9. At 40 N, in the mild wear regime, a deformation zone of about 220 μm thickness was formed beneath the surface (Figure 9a), while Mg_12_ZnY strips could coordinate with the deformation of α-Mg dendrites by bending or by forming kinks from the magnification microphotograph (Figure 9b). It was reported that the kink bands developed when compressive stress was loaded parallel to the (0001) plane—i.e., basal slippage was inhabited—and they could contribute to some extent to the ductility of the Mg97Zn1Y2 alloy [22]. When the load was increased to 100 N under the severe wear regime, a DRX zone of about 40 μm thickness was formed, followed by a plastic deformation zone of about 120 μm thickness underneath (Figure 9c). The Mg_12_ZnY strips in DRX zone were essentially as intact as in plastic deformation zone, maintaining a fiber enforcing effect, as shown in the magnification microphotograph (Figure 9d).

**Figure 10:**
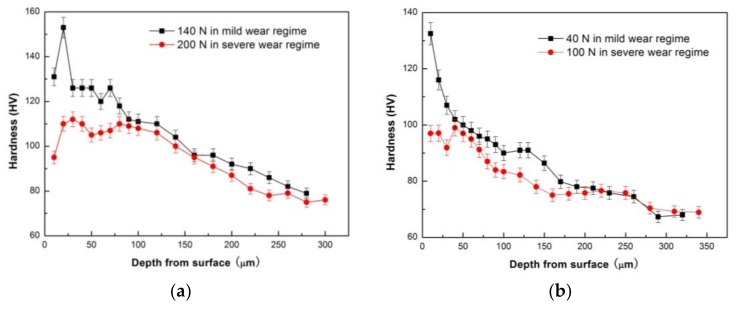
Variations in hardness with depth from the worn surface for Mg97Zn1Y2 alloy sliding at 0.8 m/s (**a**) and 3.0 m/s (**b**) before and after mild–severe wear transition.

The microstructural observation reveals that the most important change in the subsurface microstructure before and after the mild–severe wear transition is the transformation from the deformed to the DRX microstructure. It is known that Mg and its alloys have limited ductility due to their close-packed hexagonal structure, which is why the cracks are typically formed on worn surfaces under the delamination mechanism. Therefore, it is the DRX realization that improves the plastic deformation ability of the surface layer material and brings about the disappearance of cracks on the worn surfaces, even under a large plastic deformation (extruded edge). The transformation from the plastic deformed to the DRX microstructure in the surface layer material could also bring about changes to the mechanical properties accordingly, because the DRX transformation is a typical softening mechanism during the hot deformation of magnesium alloys. Therefore, the hardness distribution on the subsurfaces before and after the mild–severe wear transition were compared to identify the softening effect of DRX realization. Figure 10a,b show the variations in hardness with depth from the surface for the selected pins after sliding at 0.8 m/s and 3.0 m/s, respectively. At 0.8 m/s, it can be seen that with increasing depth, the subsurface hardness measured at 140 N decreased until the original hardness of 76 HV at a depth of 280 μm, except for the point at the depth of 10 μm. The hardness increasing above the original hardness of the studied material could be due to surface oxidation (oxide layer or MML) and strain hardening of surface layer material. The oxide layer is typically harder than magnesium alloys. The extremely high hardness within 0–20 μm could be largely influenced by MML, whereas high hardness within 20–260 μm is apparently ascribed to the strain-hardening effect. The strain-hardening effect weakens with increasing depth. However, the subsurface hardness measured at 200 N is much lower than that at 140 N, within a depth range of 0–100 μm, and the hardness curve presents a valley at 50 μm before rising a little and then decreasing to the original hardness of the alloy at 240 μm. The subsurface softening aroused by the larger load of 200 N is contrary to the strain-hardening effect, and the width of the softening zone roughly agrees with the depth of the DRX zone. This proves that the DRX softening effect occurring in the subsurface after the mild–severe wear transition from the aspect of hardness change. Similarly, at 3.0 m/s, the strain-hardening effect was also observed in the variation of subsurface hardness with depth for a pin worn at 40 N in the mild wear regime, while a softening effect was found within 40 μm in the subsurface hardness curve for a pin worn at 100 N under the severe wear regime. Consequently, it can be concluded from the aspects of microstructure evolution and subsurface hardness change that the mild–severe wear transition mechanism is the DRX softening of the surface material in the wear process.

### 3.6. Critical Surface DRX Temperature Criterion for the Mild–Severe Wear Transition

It was reported that a large equivalent shear plastic strain accumulated in the subsurfaces of magnesium alloys during dry sliding wear, even though the applied load was much less than the mild–severe wear transition load [10,11]. There are two necessary conditions for DRX realization in the subsurface, namely sufficient plastic strain and surface temperature. As plastic strain was always found to satisfy the requirements even before the mild–severe wear transition, the only condition to considered was surface temperature. Therefore, we propose a critical surface temperature criterion for the mild–severe wear transition based on the above experimental results and analyses. Once the friction-induced contact surface temperature *T_S_* reached the onset temperature of DRX *T_DRX_*, the DRX softening effect simultaneously took place in the near surface region, triggering the mild–severe wear transition. The criterion is expressed by Equation (1).

(1) $$T_{S}\geq T_{DRX}$$

The DRX dynamics point out that the DRX realization period can be expressed by a function of DRX temperature and activation energy. In the present study, it can be presumed that when mild wear transits into severe wear, most of the sliding contact period contributes to the DRX realization of the surface material, that is, the sliding contact period approximately equals the DRX realization period. Therefore, a correlation between the sliding speed and critical DRX temperature of the surface material can be established using DRX dynamics. The DRX realization period *t* can be expressed by Equation (2) using the Johnson–Mehl–Avrami–Kolmogorov (JMAK) equation [23].

(2) $$t=\frac{L}{v}\approx B\exp(\frac{Q}{RT_{DRX}})$$

where *L* and *v* are the sliding distance and sliding speed respectively, *B* the experimental constant, *Q* the apparent activation energy for DRX, and *R* the gas constant.

The values of the experimental constant *B* and the activation energy for DRX *Q* must be estimated firstly before calculating the critical surface DRX temperatures at various sliding speeds using Equation (2). For these purposes, hot compression tests of the studied alloy were carried out within a certain strain rate range. In the hot deformation process of magnesium alloys, the onset temperature of DRX mainly depends on the strain rate [24,25,26,27]. Since the worn specimen at 1.0 m/s and 140 N was at the critical state of mild–severe wear transition and its surface had a low oxygen element content (5.82%), choosing it for the evaluation of the subsurface plastic strain rate can avoid the influence of the surface oxidation layer. The distribution of equivalent plastic strain in the subsurfaces at 1.0 m/s and 140 N were measured as a function of the sliding cycle (sliding distance), using the curvature of the flow lines according to Equation (3) [27,28], and the measured average strain rate at depth of 0–15 μm was determined to be within a range of 1.0 × 10^−2^ to 8.0 × 10^−2^ s^−1^.

(3) $$\varepsilon (z)=\frac{\sqrt{3}}{3}\tan(\theta (z))$$

where *z* is the depth beneath the worn surface, and *θ* is the shear angle of the flow lines.

### 3.7. Determination of Activation Energy and DRX Temperatures 

It has been reported that most magnesium alloys experience DRX softening at temperatures of 200–360 °C when subjected to hot deformation [24,25,26,27]. Considering the high DRX temperature of Mg97Zn1Y2 alloy, in order to determine the activation energy and temperature for DRX in the subsurface of the alloy at strain rates of 1.0 × 10^−2^ to 8.0 × 10^−2^ s^−1^, hot compression tests of Mg97Zn1Y2 alloy were performed within a strain rate range of 1.0 × 10^−4^–1.0 × 10^−1^ s^−1^ and a temperature range of 300–380 °C.

**Figure 11:**
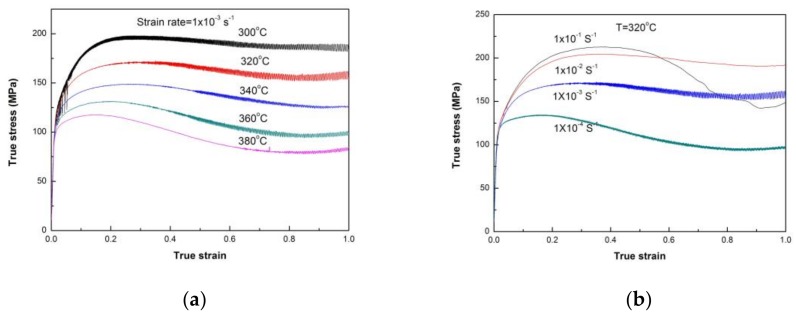
True stress-strain curves of Mg97Zn1Y2 alloy under hot compression conditions: (**a**) 300–380 °C, 1 × 10^−3^ s^−1^; (**b**) 320 °C, 1 × 10^−4^ s^−1^ × 10^−1^ s^−1^.

The true-stress–true-strain curves at the strain rate 1.0 × 10^−2^ s^−1^ and temperature 320 °C are shown in Figure 11. It can be seen from Figure 11a that when the deformation temperature is increased above 320 °C, strain softening begins to occur clearly. The great decrease of true stress at 1.0 × 10^−1^ s^−1^ occurring at strain above 0.4 in Figure 11b is not a real result, and was found to be caused by the cracking of the specimen during the hot-compressive test. The curves at 1.0 × 10^−4^–1.0 × 10^−2^ s^−1^ display the normal single peak and a concave-down appearance, i.e., after the peak the stress decreases with increasing strain due to DRX. Therefore, the strain hardening and strain softening of Mg97Zn1Y2 alloy varies with the strain rate and deformation temperature. The relation between the strain rate , flow stress *σ* and deformation temperature *T* can be described by the hyperbolic sine law proposed by Sellars and Tegart [29].

(4) $$\dot{\varepsilon }=A{\left[\sinh(\alpha \sigma )\right]}^{n}\exp(-\frac{Q}{RT})$$

**Figure 12:**
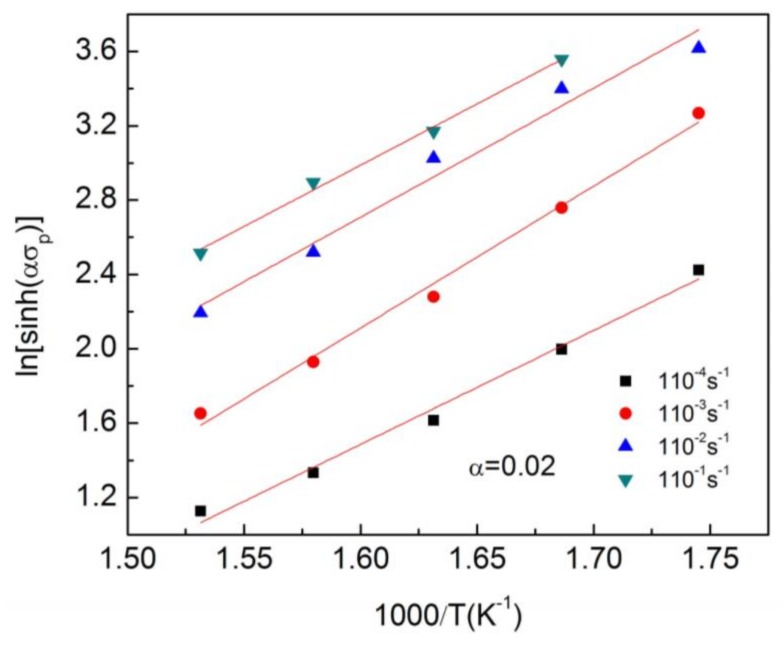
The linear relationship between ln[sinh (*ασ*)] and 1/T.

**Figure 13:**
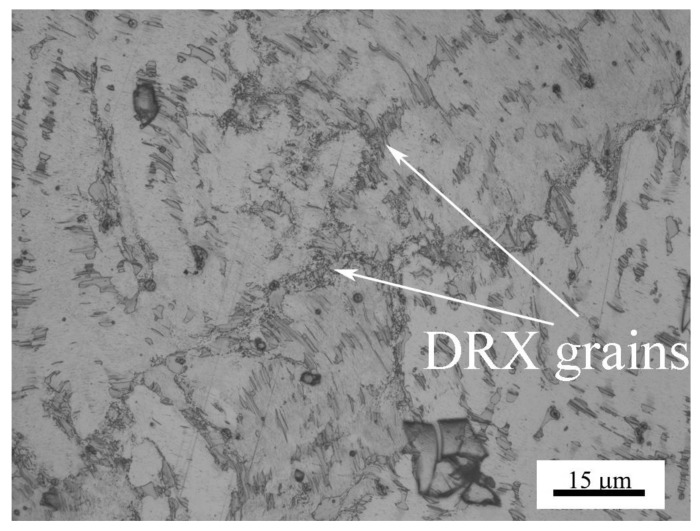
Optical microstructure of Mg97Zn1Y2 alloy hot-deformed at 320 °C and at strain rate of 1 × 10^−1^ s^−1^.

The value of *Q* was calculated to be 238.2 kJ mol^−1^ based on the slopes of lines in Figure 12. The microstructure examination of the hot deformed specimens of Mg97Zn1Y2 alloy revealed that DRX took place at 320 °C and above at strain rates of 1 × 10^−2^–1 × 10^−1^ S^−1^, as shown in Figure 13. The observed DRX temperature of Mg97Zn1Y2 alloy was very close to the DRX temperature of 325 °C for an extruded Mg96Zn1Y3 alloy containing X-Mg_12_ZnY phase, which was observed during tensile tests at strain rates of 1 × 10^−4^–1 × 10^−3^ S^−1^ by Li et al. [16]. As a result, *B* can be determined to be 4.83 × 10^−19^ s by Equation (2) using the data obtained at 1.0 m/s, namely DRX temperature 320 °C and activation energy *Q* 238.2 kJ mol^−1^. The critical DRX temperatures at other sliding speeds were subsequently calculated, as listed in Table 4. It is noted that the critical DRX temperature varies between 311.6 °C and 340.7 °C as the sliding speed is increased from 0.5 to 4.0 m/s. This variation trend apparently follows the DRX dynamics, namely that high sliding speeds make the contact surface reach a high temperature state faster than low sliding speeds, which allows DRX realization of the surface layer material at a higher temperature.

### 3.8. Evaluation of Mild–Severe Wear Transition Loads 

At a given sliding speed, the mild–severe wear transition of magnesium alloys can be characterized by the transition load. The relationship between the average surface temperature *T_S_* in sliding contact, applied load *F* and sliding speed *v* can be expressed using Equation (5), proposed by Lim and Ashby [30].

(5) $$T_{S}=T_{0}+\frac{\alpha \mu Fvl_{b}}{A_{n}K_{mp}}$$

where *T*_0_ is the temperature of the heat sink where the heat flows, it can be considered as the room temperature of 20 °C; *α* is the fraction of the heat conducted into the pin, *μ* is the coefficient of friction, *A_n_* is the nominal contact area, *l_b_* is the mean diffusion distance, and *K_mp_* is the thermal conductivity of the pin. 

**Figure 14:**
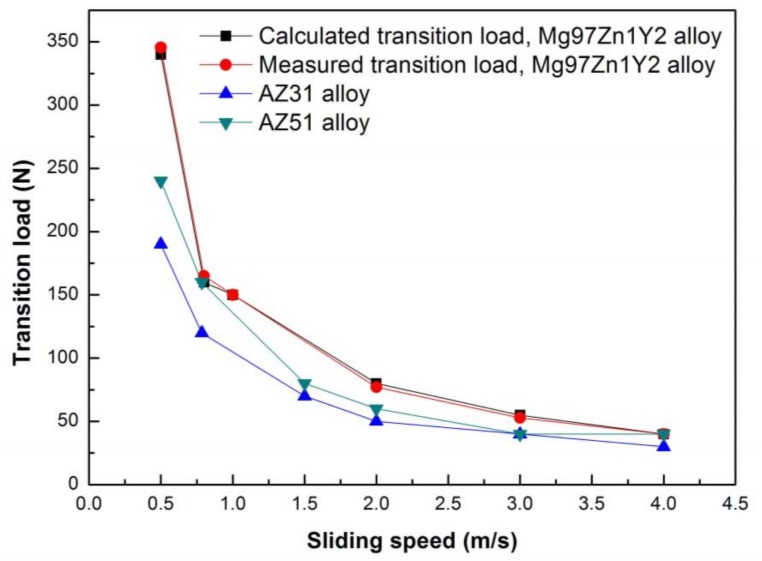
Measured and calculated transition loads of Mg97Zn1Y2 alloy at different sliding speeds.

When the sliding wear is under the critical condition of the mild–severe wear transition, an almost steady-state temperature distribution of both pin and disc can be established [8]. It is reasonable to assume that the parameters *α*, *l_b_*, *A_n_*, *K_mp_* in Equation (5) are approximate constants under different sliding speeds. Therefore, Equation (5) can be rewritten as Equation (6) by introducing a constant *K_DRX_*.

(6) $$F_{T}=\frac{\left(T_{DRX}-T_{0}\right)}{K_{DRX}\mu v}$$

(7) $$K_{DRX}=\frac{\alpha l_{b}}{A_{n}K_{mp}}$$

where *F_T_* is the transition load at sliding speed *v*, *K_DRX_* is an approximate constant relating with the testing equipment and material properties of the pin and the disc at the critical DRX state. The value of *K_DRX_* was determined to be 6.25 using the already known data of Mg97Zn1Y2 alloy sliding at 1.0 m^−1^ and 20 °C: *T_DRX_* 320 °C, *F_T_* 150 N and *μ* 0.32. The coefficients of friction varied around 0.32, except for the sliding speeds of 0.5 and 0.8 m/s at the transition loads, as listed in Table 4. At 0.5 and 0.8 m/s, the coefficients of friction were about 0.27 at the transition load of 340 N and 0.36 at the transition load of 160 N. By plugging the DRX temperatures and coefficients of friction into Equation (7), the transition loads at different sliding speeds were calculated, as shown in Figure 14. It can be seen that the calculated transition loads almost agree with the experimental results, even though a large deviation was at the sliding speed of 2.0 m/s and 2.9 N, about 3.6% of the measured transition load. Considering the assumptions in the modeling, the deviation is allowable. Therefore, the proposed surface DRX temperature criterion can be used to evaluate the mild–severe wear transition loads of Mg97Zn1Y2 alloy. In addition, the transition loads of AZ magnesium alloys such as AZ31 and AZ51 alloys at different sliding speeds are also incorporated in Figure 14 for the purpose of comparison [10,11]. Apparently, Mg97Zn1Y2 alloy has a better resistance to mild–severe wear transition within the sliding speed range of 0.5–4.0 m/s. The largest difference in the transition load between Mg97Zn1Y2 alloy and AZ alloys occurs at 0.5 m/s, about 100 N, and the difference reduces with increasing sliding speed until 4.0 m/s, at which Mg97Zn1Y2 alloy and AZ alloys almost have the same transition load.

## 4. Conclusions

From the above results and analysis, the following conclusions have been drawn.

(1) Friction and wear sliding tests were conducted within a wide load range at sliding speeds of 0.2, 0.8 and 3.0 m/s. Under the three sliding speeds, all the coefficients of friction decreased rapidly when load was not higher than 60 N or 55 N, and then decreased a little and gently within the load ranges of 60–220 N at 0.2 m/s, 60–160 N at 0.8 m/s and 55–120 N at 3.0 m/s, and finally maintained a low level with tiny fluctuations as the load was further increased.
(2) The wear-rate–load curves show a clear stage feature, i.e., at 0.2 m/s, there were two slowly increasing stages separated by 180 N, while at 0.8 m/s, there were two stages, a slow increase stage and a fast increase stage; at 3.0 m/s there were three stages, a slow increase stage, a fast increase stage and a high level stage.
(3) At the low sliding speed of 0.2 m/s, wear proceeded under the mild wear regime until the largest experimental load of 380 N, and the main wear mechanisms were oxidation + abrasion and delamination + surface oxidation. At the intermediate sliding speed of 0.8 m/s, the main wear mechanisms were oxidation + abrasion, delamination + surface oxidation under the mild wear regime and severe plastic deformation + spallation of oxidation layer under the severe wear regime. At the high sliding speed of 3.0 m/s, the wear mechanisms were delamination under the mild wear regime, and severe plastic deformation and surface melting under the severe wear regime.
(4) The wear rate and wear transition maps for Mg97Zn1Y2 alloy were established on a rectangular coordinate system with applied loads as the vertical coordinate and sliding speed as the horizontal coordinate.
(5) The mild–severe wear transition was caused by the softening effect in the surface layer material due to frictional heating-induced DRX realization.
(6) A the surface DRX temperature, the criterion was proposed for the determination of the mild–severe wear transition, and the critical surface DRX temperatures at various sliding speeds were calculated by means of DRX dynamics theory.
(7) The mild–severe wear transition loads of Mg97Zn1Y2 alloy at sliding speeds of 0.5–4.0 m/s can be calculated based on the critical surface DRX temperature criterion and the calculated values agree well with the measured ones.

## Figures and Tables

**Table 1:** Mechanical properties of Mg97Zn1Y2 alloy.

Compressive Yield Strength [MPa]	Compressive Strength [MPa]	Compressive Strain Limit [%]	Hardness [HV]
125.6	221.1	18.7	76

**Table 2:** Comparison of changes in wear rate and surface hardness between AZ, AS31 and Mg97Zn1Y2 alloys after mild-severe wear transition.

Material	Transition Load [N]	Load [N]	Wear Rate	Wear Rate Increase [%]	Surface Hardness [HV]	Surface Hardness Decrease [%]	Reference
AZ31	30	30	7.5	-	118	-	-
-	-	50	33.1	341.3	102	13.6	[10]
AZ51	40	40	6.5	-	113	-	-
-	-	60	12.94	99.1	98	13.2	[11]
AS31	60	60	13.5		104		
-	-	80	20.3	50.4	93	10.5	[17]
Mg97Zn1Y2	55	55	10.5	-	134	-	-
-	-	80	13.8	31.4	125	7.5	Present study

**Table 3:** Chemical composition analysis of worn surfaces under different sliding conditions (wt %).

Sliding Speed (m/s)	Wear Regime	Load [N]	O	Y	Zn
0.5	Mild	20	18.20	4.81	1.76
		60	17.96	5.38	2.23
		100	15.41	5.26	2.22
		180	12.25	5.56	2.21
		300	18.04	5.51	2.05
		340	9.38	5.72	2.39
		380	16.18	5.64	2.26
1.0	Mild	20	19.63	5.24	2.34
		80	11.92	5.41	2.41
		100	12.85	12.85	5.53
		140	5.82	7.21	2.8
	Severe	160	7.53	6.54	2.36
		220	6.79	6.20	2.42
		340	14.49	5.83	2.10
		380	14.17	5.59	2.28
2.0	Mild	20	15.70	5.93	2.33
		60	14.70	6.33	2.27
		80	9.37	6.13	2.58
	Severe	100	4.83	6.26	2.91
		180	3.24	7.09	2.74
		220	4.82	6.78	2.60
		260	9.29	6.41	1.90
		300	1.89	7.56	2.87
4.0	Mild	20	5.45	6.78	2.55
	Severe	60	5.02	6.31	2.47
		100	6.97	6.39	2.56
		140	3.28	6.16	2.31

**Table 4:** Calculated DRX (dynamic crystallization) temperatures, transition load and coefficient of friction of Mg97Zn1Y2 alloy under different sliding speeds.

v [m/s]	T_DRX_ [°C]	Coefficient of Friction	Measured Transition Load [N]	Calculated Transition Load [N]
0.5	311.6	0.27	340	345.6
0.8	317.3	0.36	160	165.2
1.0	320	0.32	150	150
2.0	328.6	0.32	80	77.2
3.0	336.9	0.32	55	52.8
4.0	340.7	0.32	40	40.1
